# Optimizing Budget Allocation for Digital Health Investments Using Metaheuristic Algorithms: A Cost–Impact Analysis for Public Health Systems

**DOI:** 10.3390/healthcare14111540

**Published:** 2026-06-01

**Authors:** Faruk Dayi, Aylin Erdogdu, Yusuf Esmer, Ferah Yildiz, Farshad Ganji

**Affiliations:** 1 Faculty of Economics and Administrative Sciences, Kastamonu University, 37160 Kastamonu, Türkiye; fdayi@kastamonu.edu.tr; 2 Faculty of Economics and Administrative Sciences, İstanbul Arel University, 34295 İstanbul, Türkiye; 3 Faculty of Applied Sciences, Bayburt University, 69300 Bayburt, Türkiye; yesmer@bayburt.edu.tr; 4 Faculty of Management, Kocaeli University, 41350 Kocaeli, Türkiye; ferah.yildiz@kocaeli.edu.tr; 5 Software Development Department, Istanbul Aydin University, 34295 İstanbul, Türkiye; farshadganji@stu.aydin.edu.tr

**Keywords:** health economics, digital health investment, public health system, cost–impact optimization, fuzzy logic, genetic algorithm, AICOT

## Abstract

**Background**: In the era of digital transformation, public health systems increasingly rely on digital technologies to improve accessibility, efficiency, and patient outcomes. However, policymakers face significant challenges in allocating limited resources across competing digital health investments characterized by uncertainty and dynamic impacts. **Methods**: This study introduces the Adaptive Impact–Cost Optimization Theory (AICOT), a hybrid framework integrating fuzzy logic and genetic algorithms to optimize digital health investment portfolios. The model defines the Investment Priority Score (IPS) as a function of cost, expected impact, and implementation feasibility, enabling structured evaluation under uncertainty. A fuzzy inference system with centroid-based defuzzification is used to convert qualitative assessments into quantitative scores, while optimization techniques identify optimal portfolios across different fiscal scenarios. The empirical analysis covers 15 OECD countries (2018–2024) using publicly available datasets. Sensitivity analyses assess robustness under inflation, cost shocks, and changing system priorities. **Results**: The findings show that blended investment strategies combining routine digital health tools with pandemic-oriented infrastructures yield the highest resilience-adjusted efficiency. Results remain stable across sensitivity scenarios, with pandemic surveillance consistently ranking as a top priority even under increased cost conditions. The model effectively captures cross-country heterogeneity, demonstrating adaptability to different levels of digital maturity. **Conclusions**: AICOT provides a transparent and policy-relevant decision-support framework that improves resource allocation efficiency and reduces unnecessary expenditures. These contributions support long-term financial sustainability and align with global health objectives, including Universal Health Coverage and Sustainable Development Goal 3 (Good Health and Well-being).

## 1. Introduction

Rising costs, workforce shortages, an ageing population with increasing care needs, and intensifying market competition collectively pose significant challenges to contemporary healthcare systems. Despite these pressures, consumers continue to demand higher-quality and more responsive services [1]. At the same time, health systems operate under substantial strain, making the efficient allocation and utilization of scarce healthcare resources essential to achieving value for money [2]. In response to these structural pressures, healthcare organizations are increasingly turning to digital technologies and artificial intelligence to meet consumer expectations, mitigate labor shortages, reduce operational costs, and enhance the quality of care delivery. Notably, many healthcare executives acknowledge that their institutions are not doing enough to address factors critical for long-term sustainability. Current estimates suggest that artificial intelligence, machine learning, and deep learning have the potential to generate between USD 200 billion and USD 360 billion in cost savings across the healthcare sector [1].

Digital health interventions (DHIs) employ a range of digital technologies—including digital health applications as well as information and communication technologies—to achieve specific health objectives [3]. These technologies are increasingly shaping the functioning of healthcare financing by structuring interactions among individuals, healthcare providers, and government health authorities. When appropriately designed and effectively implemented, digital solutions can streamline financing processes, enabling them to be executed with greater efficiency and accuracy. Such improvements support the overarching goal of universal health coverage by enhancing system efficiency, promoting transparency, and facilitating a more equitable allocation of resources. Well-developed digital financing tools also attract greater stakeholder engagement and adoption. These tools encompass database management systems, data analytics platforms, mobile and web applications, digital payment mechanisms, blockchain-based solutions, and artificial intelligence (AI) applications. AI-driven technologies, in particular, can perform complex tasks with ease and at scale. For example, they support the personalization of health services, the tailoring of care to individual patient needs, and the overall improvement of service quality [4].

The importance of digital health technologies continues to expand within the rapidly evolving healthcare sector. Ranging from digitally supported treatment modalities to AI-enabled diagnostic tools, these innovations aim to transform patient care, enhance clinical outcomes, and increase system-wide efficiency by delivering higher-quality health services. Given the breadth and diversity of available digital health solutions, conducting a rigorous economic assessment prior to investment is essential.

Although digital health systems offer substantial benefits, their implementation and long-term sustainability require considerable financial commitment. Decisions regarding the procurement of digital products or the development of new systems vary according to the healthcare budget, institutional priorities, and the expectations of the budget holder. A substantial initial investment is required for the development, maintenance, and ongoing support of digital health technologies [5].

Digital health interventions (DHIs) are defined as technologies with the potential to address the growing demand for healthcare services, enhance the efficiency and sustainability of health systems, and improve both the accessibility and the quality of care. The adoption and utilization of DHIs in healthcare delivery were strongly promoted during the COVID-19 pandemic, which highlighted their critical role in ensuring continuity of services and expanding access under unprecedented constraints [6]. Digital health interventions improve the efficiency and quality of care in part by reducing waste and unnecessary expenditures within healthcare systems. Despite these anticipated benefits, the evidence base regarding the cost-effectiveness of digital health tools remains limited, underscoring the need for more rigorous economic evaluations [7]. However, economically assessing the costs and outcomes of digital health technology interventions remains one of the most effective tools available to health policymakers. By demonstrating the value generated from DHI investments, economic evaluation supports the efficient allocation of scarce resources and enables more rapid and informed improvement efforts within global health systems [8].

Digital health investments have become a central pillar of modern health system strengthening strategies, driven by increasing demands for efficiency, improved care coordination, and enhanced resilience against public health emergencies. The COVID-19 pandemic highlighted critical disparities in countries’ digital readiness, demonstrating that robust electronic health records (EHRs), real-time surveillance infrastructures, artificial intelligence (AI)-supported diagnostic tools, and mobile health (mHealth) platforms are not only technological enhancements but essential components of resilient health systems. Countries that had invested strategically in these technologies prior to the pandemic were better equipped to sustain essential services, mitigate service disruptions, and implement targeted containment strategies [9,10,11,12,13].

The COVID-19 pandemic further accelerated digital adoption while exposing structural weaknesses in health information systems. Emergency conditions produce unpredictable demand surges, fluctuating technology prices, and rapidly evolving policy priorities. Countries with strong digital infrastructure, such as integrated EHRs, real-time surveillance systems, and telehealth networks, were better able to maintain essential services, manage vaccination logistics, and coordinate public health responses [14,15,16]. From a health economics perspective, resource allocation for digital technologies presents complex trade-offs. Digital tools often require substantial upfront investments in infrastructure, workforce training, interoperability, and cybersecurity [17,18]. Although long-term economic analyses suggest that digital health can generate cost savings through improved efficiency and reduced duplication, these benefits depend on context, governance maturity, and user adoption [19,20]. Many governments continue to use conventional budgeting tools such as cost-effectiveness analysis (CEA) and cost-utility analysis (CUA), which do not adequately capture qualitative uncertainties, non-linear impacts, or dynamic fiscal pressures [21]. 

Despite increased global attention, digital health investment planning remains limited. In many countries, decisions are heavily influenced by donor preferences, short-term political interests, or expert opinion rather than systematic, data-driven analysis [22]. Existing approaches rarely consider cooperation between routine digital health tools and pandemic-specific infrastructures. However, empirical evidence shows that investments in interoperable data platforms, early warning systems, and remote monitoring capabilities significantly enhance both day-to-day efficiency and emergency response capacity [15,23].

To address these gaps, this study introduces the Adaptive Impact–Cost Optimization Theory (AICOT), an integrated prioritization model combining fuzzy logic and Genetic Algorithm (GA) optimization. Fuzzy logic enables the quantitative representation of qualitative uncertainties, such as feasibility, stakeholder readiness, or perceived impact. At the same time, GA allows for the exploration of large, complex investment spaces with non-linear interactions [24,25]. AICOT evaluates digital health investments under multiple budget scenarios, capturing trade-offs among cost, expected impact, equity, and implementation feasibility.

The framework also incorporates pandemic-related considerations such as demand volatility, surveillance capacity, and infrastructural resilience. By using data from the WHO, OECD, and publicly available datasets covering 15 OECD countries between 2018 and 2024, AICOT produces ranked investment portfolios optimized for both routine and emergency conditions. This aligns with international agendas, including the UHC, SDGs, and the WHO Global Strategy on Digital Health [22,26]. Despite growing interest in digital transformation, governments face persistent challenges in allocating limited budgets across competing digital health priorities. Traditional health economic evaluation tools, such as CEA, CUA, and budget impact analysis (BIA), struggle to adequately capture the multidimensional value of digital technologies, which often produce indirect benefits, non-linear returns, and long-term system-wide effects. Moreover, uncertainty in implementation costs, workforce readiness, regulatory maturity, and adoption rates complicates the prioritization process.

To address these gaps, this study introduces the Adaptive Impact–Cost Optimization Theory (AICOT), a decision-support framework designed to guide policymakers in identifying cost-efficient, high-impact digital health investment portfolios under conditions of uncertainty. AICOT integrates economic reasoning, feasibility considerations, and dynamic system resilience requirements into a unified structure, supporting evidence-informed resource allocation. By accounting for marginal benefits, opportunity costs, and budgetary constraints, AICOT provides a health-economics-aligned, context-sensitive tool for strategic investment planning.

Importantly, AICOT is designed to be transparent and replicable. By leveraging publicly available datasets from the WHO, OECD, OSF, and other sources, the framework ensures that prioritization decisions are grounded in empirical evidence rather than intuition. It also aligns with international health policy agendas, including Universal Health Coverage (UHC), the Sustainable Development Goals (SDGs), and the WHO Global Strategy on Digital Health, providing a tool to support countries in achieving long-term health system strengthening objectives.

The primary objective of this article is to operationalize AICOT as a practical portfolio optimization framework that enables policymakers to: (i) evaluate the economic and operational value of digital health investments; (ii) incorporate uncertainty into priority-setting; and (iii) adjust investment decisions across varying fiscal and epidemiological scenarios. In doing so, the study contributes to ongoing debates in health economics concerning optimal budgeting strategies for digital transformation and the integration of resilience-focused investments into national health systems.

Despite increasing investments in healthcare digitization, many public health systems still lack practical tools for allocating limited budgets across competing priorities. Traditional budgeting methods often focus only on immediate costs and may overlook implementation feasibility, uncertainty, and long-term resilience benefits. As a result, investment decisions can become fragmented and inefficient.

To address this challenge, this study develops the Adaptive Impact Cost Optimization Theory (AICOT), a practical framework designed to improve digital health investment efficiency under budget constraints. The model evaluates competing investment alternatives according to cost, expected impact, and implementation feasibility under changing fiscal conditions.

Accordingly, this study tests three main hypotheses. First, adaptive optimization approaches are expected to outperform static budgeting methods. Second, mixed portfolios combining routine and emergency digital technologies are expected to generate higher resilience-adjusted efficiency. Third, investment priorities are expected to vary significantly across baseline, fiscal tightening, and pandemic surge scenarios.

Following this introduction, Section 2 presents literature; Section 3 presents literature reviews, Section 4 presents methods; Section 5 solves the pattern and analyzes the results; Section 6 presents results and discussion; and Section 7 presents conclusion, policy implications, and suggestions for future research.

## 2. Conceptual Framework

The rapid digital transformation of health systems over the past two decades has generated a substantial body of literature encompassing health economics, digital technology adoption, and computational decision-support models. This section synthesizes key strands of this research, organizing insights into three interrelated themes: (i) digital health and system performance, (ii) economic evaluation for digital technologies, and (iii) the emerging role of fuzzy logic and metaheuristic optimization in health policy modeling.

### 2.1. Digital Health and System Performance

A vast body of literature demonstrates the transformative potential of digital technologies for enhancing health system efficiency, accessibility, and quality. In bibliometric analyses, the terms *telemedicine* and *telehealth* emerge as the most frequently used keywords within the digital health domain. Conversely, the concepts eHealth, digital health, and mHealth are also consistently emphasized in the literature, reflecting the breadth and evolving scope of digital health research [6]. Electronic health records (EHRs), telemedicine, AI-supported diagnostics, and mobile health platforms are among the most widely studied domains. EHR systems, for example, are associated with reductions in medical errors, improved care coordination, and enhanced surveillance capacity [19,27,28]. In many countries, the implementation and maintenance of electronic health records (EHRs) entail substantial financial investments. Conducting evaluations of publicly funded EHR initiatives can provide critical insights into whether healthcare resources are being utilized efficiently and effectively [29]. Interoperability, the ability of systems to exchange and interpret data, is repeatedly identified as a key determinant of EHR impact [30]. Despite significant global investment, interoperability remains uneven, particularly in low- and middle-income countries (LMICs), where limited infrastructure and digital governance reduce system-wide value. The rapid proliferation of digital health technologies over the past two decades has fundamentally reshaped the structure and functioning of health systems worldwide. A wide range of empirical studies demonstrates that digital transformation contributes significantly to improving efficiency, care coordination, patient safety, and population-level outcomes. Electronic health records (EHRs), telemedicine platforms, mobile health (mHealth) applications, and artificial intelligence (AI) decision-support systems represent the core pillars of this transformation [31].

Telemedicine has emerged as a significant field of research, especially since the COVID-19 pandemic. Systematic reviews indicate that telehealth enhances access for rural populations, reduces unnecessary hospital admissions, and lowers healthcare costs when integrated effectively into clinical pathways [32]. Moreover, telemedicine adoption surged during pandemic lockdowns, demonstrating system-level benefits such as reduced burden on emergency departments and improved continuity of care.

Numerous studies have demonstrated that AI-based interventions can achieve clinical outcomes comparable to standard care, while also producing measurable improvements in lifetime quality-adjusted life years (QALYs) by reducing the risks of conditions such as stroke or atrial fibrillation [33]. AI-supported diagnostics, predictive analytics, and clinical decision-support tools have also attracted growing scholarly interest. AI systems demonstrate superior or comparable performance to clinicians in fields such as radiology, dermatology, and pathology [34]. Despite these advances, concerns persist regarding algorithmic bias, data representativeness, ethical oversight, and system integration [35]. Scholars emphasize that AI’s economic benefits, such as reduced diagnostic delays, improved workflow efficiency, and decreased readmission rates, depend heavily on the strength of underlying data infrastructures and governance capacity [19,36].

The economic literature notes that while AI may reduce diagnostic costs in the long run, up-front investments in infrastructure, training, validation, and cybersecurity are substantial. Nevertheless, concerns persist regarding quality control, regulatory frameworks, reimbursement mechanisms, and digital exclusion among older adults or individuals with limited digital literacy [9,10,11,37,38,39,40].

### 2.2. Economic Evaluation for Digital Health Technologies

The most commonly utilized terms in health economics include cost-effectiveness, health economics, economic evaluation, cost analysis, and cost–benefit analysis [6]. Economic evaluation tools, including CEA, CUA, cost–benefit analysis (CBA), and budget impact analysis (BIA), form the core of health economics methodology. These tools provide structured frameworks for comparing interventions based on incremental cost and health benefit. However, their application to digital health poses unique methodological challenges [41,42,43]. First, digital technologies often produce indirect benefits such as workflow efficiency, data standardization, or real-time surveillance, which do not translate easily into traditional health outcome metrics like QALYs or DALYs. Second, digital interventions generate significant fixed costs and network effects that alter cost curves in nonlinear ways [19,44]. Third, digital tools interact strongly with contextual factors (e.g., infrastructure, regulatory frameworks), resulting in highly variable cost-effectiveness across settings [45]. As a result, economic evaluations of digital health frequently produce inconsistent findings. For example, some randomized trials have demonstrated that telemedicine reduces costs associated with chronic disease management [21,46]. Similarly, studies of EHR adoption demonstrate efficiency gains in high-capacity systems but limited returns in limited environments. This is because these technologies have high indirect impacts and their marginal benefits emerge over the long term. The health economics literature emphasizes the need for a broader framework that considers the opportunity costs, marginal returns, and budgetary impact of digital investments.

In a survey of 200 executives from global healthcare institutions, 75% reported that while they prioritize digital transformation, they do not allocate sufficient resources to this area and often fail to integrate it into strategic planning. Respondents noted that rising healthcare costs under current macroeconomic conditions, coupled with budget constraints, hinder investments in digitalization and artificial intelligence (AI) applications. Consequently, healthcare systems face significant challenges in technological modernization.

The primary objective of digitalization and AI integration in healthcare is to fundamentally reorganize institutional operations, foster continuous innovation, and develop capabilities that generate tangible business value, such as enhanced patient acquisition, improved clinical outcomes, operational efficiency, and workforce retention. However, technologies that enable electronic health records (EHRs) alone often fail to create new value because they disrupt existing processes and fail to drive sufficient innovation in clinical workflows. From this perspective, redesigning clinical workflows and care delivery models is essential to fully realize the benefits of healthcare technology. For instance, optimizing workflows can yield time savings of 15% to 30% over a 12 h shift, potentially bridging the nursing workforce gap by up to 300,000 inpatient nurses. Moreover, cloud-based systems enhance data accessibility and quality, thereby supporting both patient- and clinician-facing applications. AI has the potential to impact all aspects of healthcare, from clinical operations to care delivery and contracting. Effective utilization of AI applications necessitates collaboration with data science teams to ensure that insights are accurately interpreted and applied [1].

Despite this evidence, most governments lack structured frameworks for prioritizing pandemic-related digital investments. Health economists highlight the absence of tools that integrate epidemiological uncertainty, dynamic resource requirements, and long-term system resilience. This gap underscores the pressing need for adaptive investment prioritization models that can strike a balance between routine care delivery and emergency preparedness within budgetary constraints. The impact of digital surveillance systems and AI-based early-warning technologies on health system resilience during pandemics is well documented, with countries exhibiting higher digital maturity consistently achieving better health and economic outcomes.

### 2.3. Fuzzy Logic and Metaheuristic Optimization in Health Policy Modeling

Fuzzy logic allows decision-makers to incorporate linguistic variables (e.g., “high feasibility,” “moderate impact”) into structured analytic models [47,48]. Fuzzy logic has been applied in healthcare system research for tasks such as disease risk prediction, hospital performance evaluation, and prioritization of resource allocation [49]. Its ability to model qualitative uncertainties makes it suitable for evaluating digital health investments where expert perceptions and contextual factors matter. Metaheuristic optimization (such as Genetic Algorithms (GA), Particle Swarm Optimization (PSO), and Grey Wolf Optimization (GWO)) is widely used for exploring large, complex decision spaces. Although rarely applied to digital health investments, these techniques are commonly used in engineering, logistics, and operations research. A growing body of literature applies metaheuristics to healthcare problems, including hospital scheduling, epidemic modeling, and resource allocation. Because digital health investments involve high uncertainty, traditional deterministic decision-making tools are inadequate. Methods such as uncertainty-sensitive multi-criteria decision analysis (MCDA), fuzzy logic, and adaptive budgeting are prominent in the literature [20,50].

Hybrid models combining fuzzy logic and GA, often termed fuzzy GA systems, have shown strong performance in complex decision environments. These models integrate fuzzy inference with GA-based search, enabling adaptive learning. Applications include environmental management, supply chain optimization, and financial portfolio selection [51,52]. Their potential for health policy, particularly digital health investment optimization, remains underexplored. This study aims to fill these gaps with the AICOT model. Given the multi-dimensional, uncertain, and dynamic nature of digital health investment decisions, fuzzy–GA hybrid methods offer a powerful toolset. They allow policymakers to evaluate alternatives based on ambiguous criteria, incorporate multiple objectives, and adapt solutions under varying constraints.

### 2.4. The Adaptive Impact–Cost Optimization Theory (AICOT)

Another persistent challenge in health investment modeling is uncertainty stemming from incomplete data, subjective judgments, and dynamic policy contexts. Fuzzy logic, introduced by Zadeh (1965) [47], offers a mathematical framework for handling imprecision by representing linguistic variables (e.g., low cost, high impact, feasible) as degrees of membership rather than binary states [28]. In health-policy modeling, fuzzy systems can translate expert qualitative assessments into quantitative parameters usable by optimization algorithms [29]. For example, a fuzzy inference system can evaluate the “priority level” of a digital health project by combining fuzzy sets for cost, impact, and feasibility. Integrating fuzzy logic with GA, therefore, creates a hybrid metaheuristic approach that reflects both computational rigor and human judgment. Such hybridization aligns with the broader trend toward explainable artificial intelligence (XAI) in public decision making, ensuring that algorithmic recommendations remain interpretable to policymakers [30].

Responding to these challenges, the present research introduces the AICOT, a novel theoretical framework that unifies fuzzy inference modeling with metaheuristic optimization. AICOT conceptualizes digital health budgeting as a multi-objective adaptive system where investment decisions evolve dynamically in response to fiscal constraints and contextual shifts.

The framework posits that optimal allocations arise not from the static maximization of efficiency, but from an iterative balancing of impact, cost, and feasibility across diverse policy scenarios. By embedding GA within a fuzzy decision layer, AICOT enables adaptive learning from simulated outcomes, making the optimization resilient to shocks such as pandemics or economic downturns.

In practical terms, the AICOT model operationalizes a decision-support system that can:

1. Integrate quantitative and qualitative data from open-access sources (WHO, OECD, OSF, Kaggle).
2. Generate ranked investment portfolios under varying budget ceilings.
3. Conduct sensitivity analyses to test robustness against inflationary or policy shocks.
4. Provide visual trade-off analyses for multi-stakeholder negotiation.

Through these capabilities, AICOT transcends the limitations of existing frameworks by delivering an adaptive, transparent, and evidence-based mechanism for prioritizing digital health investments.

### 2.5. MATLAB R2021b and Python 3.12-Based Implementation of AICOT 

To operationalize the proposed AICOT, this study employs a hybrid computational environment integrating MATLAB MathWorks (Natick, MA, USA) R2021b and Python Software Foundation (Wilmington, DE, USA) 3.12. MATLAB R2021b serves as the primary platform for developing the Fuzzy Inference System (FIS), scenario simulation, and visualization, providing the analytical backbone for translating qualitative expert judgments into quantifiable decision parameters. Using the MATLAB R2021b Fuzzy Logic Toolbox, linguistic variables such as Cost (Low, Medium, High), Impact (Low, Moderate, High), and Feasibility (Feasible, Conditional, Risky) are encoded into membership functions that capture uncertainty and contextual variability.

The MATLAB R2021b-based framework enables the modeling of multidimensional relationships between cost and impact under different budget constraints. Through the Rule Editor and Surface Viewer tools, expert-defined fuzzy rules are transformed into a set of dynamic inference surfaces that estimate the Normalized Priority Score (NPS) for each investment alternative. These fuzzy outputs are exported as structured numerical datasets and subsequently integrated into a Python 3.12-based GA engine using data exchange protocols (e.g., .mat to .csv conversion).

This hybrid pipeline allows for iterative interaction between fuzzy logic reasoning and metaheuristic search. MATLAB R2021b continuously refines the priority scores through simulation of different policy environments, such as inflation shocks or equity-oriented weighting, while Python 3.12’s GA module explores the optimal investment combinations within the feasible budget space. The resulting framework is thus both adaptive and transparent, capable of learning from previous iterations and recalibrating its optimization trajectory as system parameters evolve [47,53]. Furthermore, MATLAB R2021b’s computational robustness supports sensitivity analysis and visualization of cost impact trade-offs through heat maps, Pareto fronts, and spider charts. These graphical outputs enhance interpretability, allowing policymakers to intuitively grasp the trade-offs between competing priorities. By coupling MATLAB R2021b’s fuzzy inference and visualization capabilities with Python 3.12’s evolutionary optimization engine, the AICOT implementation provides a comprehensive, end-to-end decision-support system that bridges theory and practice in digital health budgeting.

### 2.6. Related Literature and Research Gap

Previous studies show that digital health technologies can improve efficiency, accessibility, and emergency preparedness in healthcare systems. Investments such as electronic health records, artificial intelligence diagnostics, mobile health platforms, and surveillance systems have become increasingly important, especially after the COVID-19 pandemic.

However, most previous studies examine these technologies individually rather than as competing alternatives within a limited public budget. In addition, conventional economic evaluation tools often struggle to reflect uncertainty, feasibility constraints, and rapidly changing crisis conditions. Therefore, an important research gap remains for integrated models that can prioritize digital health investments across multiple fiscal scenarios.

## 3. Literature Review

Table 1 summarizes the key findings from prior health economics and systems research relevant to optimizing digital health budgets.

Each study highlights different aspects of digital health investments, such as pandemic management, cost–impact analysis, AI applications, mobile health, and budgeting under uncertainty, providing empirical and methodological support for the assumptions and approaches used in the AICOT framework. In this way, the table demonstrates the evidence base underpinning AICOT’s design and decision-making processes:

Across the reviewed domains, five significant gaps emerge:

❖ Limited integration of routine digital health tools with pandemic-specific infrastructures in investment planning.
❖ Insufficient methods for handling qualitative uncertainty in digital health evaluations.
❖ Narrow economic evaluation frameworks that fail to capture multi-dimensional impacts (equity, feasibility, scalability).
❖ Lack of adaptive, scenario-based tools for policy decision making under volatile conditions.
❖ Absence of hybrid fuzzy–GA models for optimizing digital health investment portfolios.

The AICOT framework directly addresses these gaps by combining fuzzy inference with GA optimization to evaluate digital health portfolios across varied budget scenarios and pandemic conditions.

Despite the extensive use of fuzzy logic and genetic algorithms in healthcare investment and decision-making problems, most existing studies remain primarily descriptive and optimization-oriented, with limited emphasis on integrative scenario-based uncertainty modeling. In contrast, the proposed AICOT (Adaptive Impact Cost Optimization approach) framework introduces a hybrid structure that simultaneously incorporates expert judgment and stochastic optimization under multiple macroeconomic conditions. This distinguishes the present study from prior approaches by enhancing both methodological adaptability and decision robustness under crisis scenarios such as economic recession and pandemic surges.

## 4. Methods

This study applies the Adaptive Impact–Cost Optimization Theory (Adaptive Impact Cost Optimization approach) as a decision-support framework for healthcare policymakers. The model combines a structured uncertainty-based scoring system with an optimization procedure to identify the most efficient combination of digital health investments under budget constraints.

In practical terms, the first stage scores each investment according to cost, expected impact, and implementation feasibility. The second stage tests alternative budget combinations and selects the portfolio generating the highest total benefit within available financial limits.

The methodological framework of this study is designed to remain fully accessible to health policy and health economics reviewers. This study develops and applies the Adaptive Impact Cost Optimization Theory (AICOT), an integrated decision-support framework designed to optimize national-level digital health investment portfolios under uncertainty. AICOT combines a fuzzy inference system (FIS) to represent ambiguity in cost, expected impact, and implementation feasibility, with a Genetic Algorithm (GA)-based optimization engine to identify cost-effective investment combinations across varying fiscal scenarios.

Although exhaustive enumeration is computationally feasible for the current five-category illustrative application, the genetic algorithm was selected because the framework is intended for future expansion involving additional investment categories, multi-period planning, country-specific constraints, and stochastic scenarios. In the present setting, both exhaustive search and GA generate equivalent optimal solutions.

The objective function maximizes priority-adjusted efficiency by evaluating IPS relative to required investment cost. Within the optimization procedure, candidate portfolios were compared according to total weighted IPS generated per unit of available budget. This formulation was selected because policymakers often seek to maximize impact under fiscal constraints. Alternative formulations based on absolute IPS maximization and minimum-cost threshold selection were also examined, yielding directionally consistent portfolio rankings.

In this study, AICOT is conceptualized as an integrated decision-support model rather than a fully established theoretical system, designed to facilitate optimization under uncertainty.

### 4.1. Indicator Construction

The study sample consists of 15 OECD countries, selected based on (i) availability of digital health expenditure data, (ii) reporting of pandemic-related digital infrastructure indicators, and (iii) completeness of health system performance metrics. Five Digital health investment categories were evaluated:

❖ Electronic Health Records (EHR) systems;
❖ AI-based diagnostic tools;
❖ Digital therapeutics (DTx);
❖ Mobile health (mHealth) platforms;
❖ Pandemic-specific infrastructures, including real-time epidemiological surveillance systems and tele-epidemiology and digital contact-tracing tools.

Each digital health investment category is assessed through a structured economic lens incorporating:

❖ Cost considerations, including capital expenditures, operational costs, and long-term maintenance.
❖ Expected health system impact, such as efficiency gains, improved service continuity, and contributions to routine and emergency preparedness.
❖ Budget impact, examining how each investment influences total health expenditure under varying fiscal conditions.

This economic foundation ensures that the model aligns with core principles in health economics, such as opportunity cost, marginal benefit, and allocative efficiency.

### 4.2. Fuzzy Modelling and Genetic Algorithm Optimization

An FIS was employed to represent uncertainty inherent in rapid technological evolution, inconsistent cross-country reporting, unpredictable pandemic conditions, and dynamically shifting costs and supply constraints. Linguistic variables were defined for cost (low/medium/high), impact (low/moderate/high), and feasibility (difficult/moderate/easy). Triangular membership functions were applied to each variable to operationalize expert-like reasoning. A fuzzy rule base (*n* = 27 rules) was constructed to generate a composite Investment Priority Score (IPS) for each digital health alternative. Defuzzification used the centroid method, producing normalized priority scores ranging from 0 to 1.

To identify a cost-efficient investment budget, a Genetic Algorithm was implemented with the following configuration:

❖ Population size: 200.
❖ Selection: tournament selection.
❖ Crossover: single-point crossover (rate = 0.8).
❖ Mutation: random resetting (rate = 0.05).
❖ Termination: convergence threshold or maximum 500 generations.

For methodological transparency, the triangular membership functions were parameterized on a normalized [0, 1] scale as follows: Low = (0.00, 0.00, 0.50), Medium = (0.25, 0.50, 0.75), and High = (0.50, 1.00, 1.00). These breakpoints were applied consistently across cost, expected impact, and implementation feasibility variables. Additional robustness checks using alternative parameter values produced broadly stable rankings.

The objective function was defined as:

(1) $$Maximize\frac{\sum IPS_{i}\cdot x_{i}}{\sum Cost_{i}\cdot x_{i}}$$

(2) $$\mathrm{subject}\;\mathrm{to}:\;\sum Cost_{i}\cdot x_{i}\leq Budget$$

where $x_{i}$ indicates whether an investment category is selected (binary decision variable). Because healthcare investment decisions frequently involve incomplete information and uncertain outcomes, a structured scoring system was used. Each investment category was evaluated according to low, medium, or high cost, expected benefit, and implementation feasibility. These qualitative assessments were then converted into numerical priority scores to support objective comparison.

After calculating Investment Priority Scores, an optimization method was used to test many possible budget combinations. The purpose was to identify the portfolio that provides the highest total benefit without exceeding the available budget. This allows policymakers to compare alternative spending strategies under different fiscal conditions.

The fuzzy inference mechanism is formalized through the construction of an Investment Priority Score (IPS), which represents the composite desirability of each digital health *investment* alternative under uncertainty. In this context, IPS is explicitly defined as a multidimensional functional relationship of the form:

*IPS_i_* = *f*(*Cost_i_*, *Impact_i_*, *Feasibility_i_*) (3)

where each component is normalized and expressed through linguistic variables within the fuzzy inference system. Rather than a simple linear aggregation, this formulation captures the nonlinear interactions between cost efficiency, expected system-wide impact, and implementation feasibility. By embedding these criteria into a rule-based structure, the IPS reflects expert-like reasoning, allowing trade-offs such as high-impact but costly technologies to be evaluated in a balanced and policy-relevant manner. This explicit formulation strengthens the theoretical foundation of the AICOT model and clarifies how qualitative judgments are systematically transformed into quantitative decision scores. Following rule aggregation, the fuzzy output is transformed into a crisp Investment Priority Score through the centroid (center of gravity) defuzzification method. This approach calculates the weighted average of the output membership function, where each possible outcome is weighted by its degree of membership. Mathematically, this is expressed as the ratio of the first moment of the aggregated membership function to its total area. The centroid method is particularly appropriate in this study because it preserves the influence of all activated rules, ensuring that no single criterion disproportionately dominates the final outcome. In contrast to alternative defuzzification techniques (e.g., maximum membership methods), the centroid approach produces a smooth and continuous output space, which is essential for optimization processes such as Genetic Algorithms. This ensures a consistent and interpretable transition from qualitative rule-based reasoning to quantitative optimization inputs, thereby strengthening the methodological coherence of the model.

To avoid null portfolio solutions and zero-denominator cases, an additional feasibility constraint was imposed requiring at least one investment category to be selected:

∑xi ≥ 1(4)

Accordingly, all feasible solutions represent economically meaningful investment portfolios.

The Investment Priority Score (IPS) should be interpreted as a composite economic desirability indicator rather than a direct monetary return measure. It summarizes the relative attractiveness of each digital health investment by jointly considering expected impact, implementation feasibility, and cost burden. Accordingly, higher IPS values indicate investments with stronger expected policy value under constrained public budgets.

Three sets of policy-relevant fiscal scenarios were simulated: baseline (pre-pandemic conditions), fiscal tightening (reduced national budgets due to an economic downturn), and Pandemic surge (elevated demand for digital triage, surveillance, and remote care services). Additionally, sensitivity analyses tested “±10–25% inflation in digital technology costs”, “disruptions in global supply chains”, “reduced or expanded interoperability capacity”, and “changes in health workforce digital literacy”. These analyses evaluated the robustness of AICOT-generated portfolios under realistic conditions of uncertainty.

The Adaptive Impact Cost Optimization Theory (AICOT) model generates several decision-support outputs designed to guide policymakers in allocating limited digital health budgets under uncertainty. Each output offers a distinct perspective on how various investment combinations impact system performance, economic efficiency, and pandemic preparedness.

### 4.3. Ranked Digital Health Investment Budget

AICOT produces rank-ordered portfolios that identify which combinations of digital health interventions offer the highest value relative to their cost. These rankings are based on composite Investment Priority Scores (IPSs), the budget constraints defined for each scenario, and the optimization results obtained from the Genetic Algorithm. The model returns both the single most optimal budget and a set of near-optimal alternatives, allowing policymakers to compare strategies under varying constraints or political priorities. This ranking supports transparent and evidence-informed decision making by showing which digital tools deliver the most significant marginal benefit per unit of expenditure.

AICOT generates cost–impact trade-off curves that illustrate how incremental changes in the investment mix affect overall system performance. These curves highlight:

❖ Diminishing returns on high-cost technologies;
❖ The point at which additional spending yields limited health system improvement;
❖ Cost thresholds beyond which efficiency declines;
❖ The relative contribution of each technology to routine care and emergency response capacity.

From a health economics perspective, helping governments determine whether proposed digital investments fall on, below, or above the frontier. For each digital health alternative, the model calculates a scenario-specific priority score that reflects its attractiveness under different fiscal contexts. These scores integrate fuzzy evaluations of cost volatility, expected impact on service delivery and preparedness, and feasibility given existing system capacity. For example: (1) Under fiscal tightening, low-cost and high-feasibility interventions (e.g., mHealth platforms) receive higher scores, and (2) under pandemic surge scenarios, technologies that strengthen surveillance and remote diagnostics gain priority. These scenario-adjusted priority scores enable governments to tailor investment plans to real-world conditions, rather than relying on static, one-size-fits-all rankings.

The model produces resilience-adjusted efficiency estimates that compare the performance of budgets optimized for routine care versus those designed to handle pandemic shocks. These estimates quantify:

❖ How well each budget maintains essential services during disruptions;
❖ The added value of including pandemic-specific digital infrastructure;
❖ The economic penalties associated with underinvestment in preparedness;
❖ Efficiency losses when digital infrastructure is insufficiently diversified.

By incorporating resilience into the efficiency calculation, AICOT moves beyond conventional cost-effectiveness analysis, providing a more realistic assessment of long-term value. Budgets that integrate both routine digital tools and emergency-ready infrastructures generally demonstrate higher resilience-adjusted efficiency, confirming the importance of blended digital investment strategies.

## 5. Solve the Pattern and Analyze the Results

This section presents the core dataset constructed for the Adaptive Impact Cost Optimization Theory (AICOT) model, explaining how the underlying patterns were identified, formalized, and analyzed. By solving the structural relationships within the data cost variability, digital adoption maturity, and resilience-related performance differences, the model derives robust, scenario-dependent investment priorities. The purpose of this section is to ensure conceptual transparency and methodological reproducibility for peer reviewers.

The fuzzy logic component constitutes the qualitative reasoning layer of AICOT. Implemented in MATLAB R2021b, it converts uncertain and expert-based judgments into continuous quantitative metrics. Three input variables, Cost, Impact, and Feasibility, are expressed through linguistic categories (e.g., Low, Medium, High) and mapped to membership functions calibrated by subject-matter experts.

A rule base comprising twenty-seven conditional statements determines the output variable, Priority. These rules emulate expert reasoning; for instance, high impact combined with low cost and high feasibility yields a very high priority, while moderate impact and high cost produce a medium priority. MATLAB R2021b’s Mamdani inference engine aggregates these rules and produces a defuzzified output through the centroid method.

### Data Set for Analysis

Data were compiled from publicly accessible international repositories, including: World Bank (Washington, DC, USA) World Development Indicators, World Health Organization (WHO) (Geneva, Switzerland), Global Health Observatory, Organization for Economic Co-operation and Development (OECD) (Paris, France) Health Statistics, Open Science Framework (OSF) (Charlottesville, VA, USA) public datasets, Kaggle Public Datasets (Kaggle Inc., San Francisco, CA, USA), and National digital health strategy documents (2018–2024) [60,61,62,63,64,65].

The resulting Normalized Priority Score (NPS) values, scaled between 0 and 1, serve as the quantitative expression of each project’s composite desirability. These values are subsequently transmitted to the Python 3.12 environment for optimization. The design ensures that uncertainty often neglected in deterministic economic models is systematically represented within the analytical process.

The dataset includes the following variable groups:

Cost Structure Variables:

❖ Capital expenditure for major digital health technologies;
❖ Operational expenditure (annualized);
❖ Implementation overhead (training, regulatory compliance, integration);
❖ Pandemic-period cost acceleration (supply chain disruption, inflation shocks).

Impact Variables:

❖ Efficiency gains (reduction in patient wait times, administrative costs);
❖ Diagnostic accuracy improvements (AI and decision-support systems);
❖ Emergency response capabilities (surveillance, real-time data flows);
❖ Contribution to Universal Health Coverage (UHC) and SDG readiness.

Feasibility Variables:

❖ Infrastructure maturity;
❖ Workforce digital literacy;
❖ Policy readiness and interoperability;
❖ Vendor ecosystem stability.

Patterns were analyzed to ensure that each variable exhibits realistic covariance structures—for example, countries with high EHR adoption generally demonstrate greater interoperability and lower marginal costs for integrating new tools. The refined dataset was specifically designed to support pattern extraction, fuzzy modeling, and Genetic Algorithm optimization. The indicators reported in Table 2 were constructed using annual OECD observations covering the 2018–2024 period. First, country-level annual data were collected for each variable. Second, missing observations were linearly interpolated where necessary. Third, all variables were normalized to a 0–100 scale using min-max transformation. Finally, multi-year averages were calculated to reduce temporary volatility and generate stable model inputs.

Table 2 presents the dataset for the AICOT Model of OECD countries.

Analysis of country-level indicators (Table 2) reveals substantial heterogeneity in digital health readiness across OECD countries. Nations such as Sweden, the Netherlands, and the United Kingdom exhibit the highest EHR coverage (>98%) and surveillance system readiness (>0.88), which correlates with elevated pandemic digital response scores. In contrast, countries such as Japan and Italy exhibit lower adoption rates for AI diagnostics and mHealth platforms, suggesting a limited capacity to rapidly scale digital health interventions during public health emergencies. Average intervention costs also vary considerably, reflecting differences in the size of the health system, labor costs, and technological infrastructure.

Table 3 (Fuzzy Input Dataset) presents the quantified costs, expected impacts, and feasibility of five key digital health investments.

The dataset used in Table 3 was constructed through a multi-stage process. First, raw indicators were collected from OECD health expenditure and macroeconomic databases. Second, these indicators were normalized and transformed into linguistic variables using predefined fuzzy membership functions. Finally, expert evaluations were incorporated to adjust scenario weights under normal, recession, and pandemic conditions. This hybrid structure ensures consistency between quantitative data and expert-driven assessments.

AI diagnostics and pandemic surveillance achieve the highest expected impact scores (0.90 and 0.95, respectively), but differ in cost and feasibility, reflecting differences in implementation complexity. Mobile health platforms and digital therapeutics exhibit lower costs and higher feasibility, suggesting that although they are easier to deploy, their direct contribution to pandemic preparedness is comparatively limited. This structured input forms the basis for calculating Investment Priority Scores using fuzzy inference.

Table 4 (The IPS results) reveals fuzzy output (Investment Priority Scores).

The IPS results (Table 4) directly translate the fuzzy inputs into actionable priority rankings. Pandemic surveillance and AI diagnostics emerge as the top priorities (0.92 and 0.88), confirming their critical role in enhancing emergency preparedness. EHR systems and mobile health platforms remain important for routine operational efficiency, while digital therapeutics rank lower in priority due to relatively minor impact on system resilience. These scores provide a quantitative basis for portfolio optimization under varying fiscal scenarios.

Table 5 demonstrates an optimal digital health investment portfolio under three budget scenarios.

The optimization results further show that no single technology dominates all scenarios. Under normal fiscal conditions, balanced portfolios combining routine and advanced digital tools perform best. Under budget constraints, lower-cost and easier-to-implement technologies become more attractive. During crisis periods, surveillance systems and AI-supported diagnostics gain the highest strategic importance. Optimized investment portfolios (Table 5) demonstrate the practical application of AICOT for decision making under different budgetary conditions. Under the baseline scenario (USD 600 million), a balanced combination of EHR, AI diagnostics, and mHealth achieves the highest overall system efficiency and resilience. Fiscal tightening prioritizes lower-cost, high-feasibility investments (mHealth + EHR), resulting in reduced total IPS (1.60). During pandemic surge conditions, portfolios emphasize resilience-critical investments, particularly pandemic surveillance and AI diagnostics, yielding the highest total IPS of 2.61. These outcomes highlight the importance of scenario-adaptive allocation strategies that strike a balance between routine efficiency and emergency preparedness.

**Figure 1:**
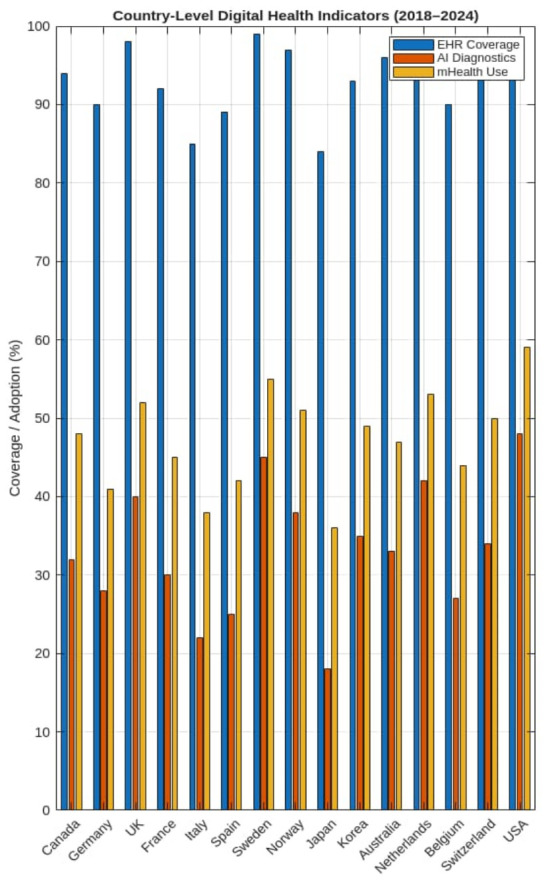
Country-level digital health readiness indicators (2018–2024) (researchers’ findings).

Figure 1 visualizes key digital health indicators from Table 1, including EHR coverage, AI diagnostics adoption, mHealth utilization, and surveillance system readiness across 15 OECD countries. The graph highlights that Sweden, the Netherlands, and the United Kingdom consistently outperform other countries across all indicators, reflecting high readiness for both routine digital health operations and pandemic response. In contrast, countries such as Japan and Italy exhibit lower adoption rates for AI diagnostics and mHealth platforms, indicating potential gaps in their capacity for rapid digital deployment during health emergencies.

**Figure 2:**
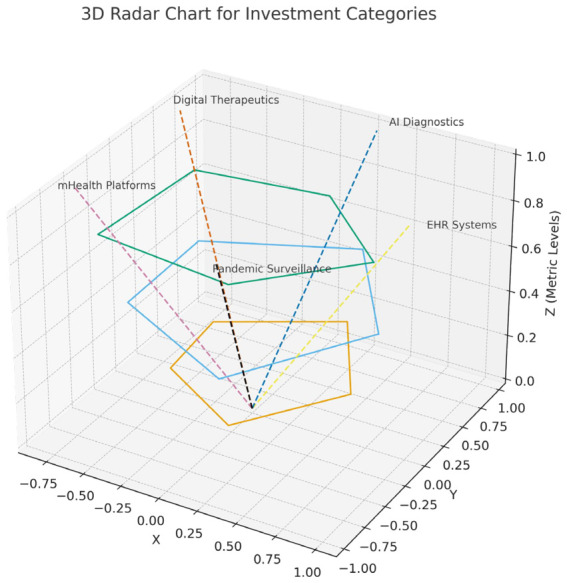
Fuzzy input scores for digital health investment categories (Researchers’ findings).

Figure 2 illustrates the normalized cost, expected impact, and feasibility scores for five investment categories (Table 3).

AI diagnostics and pandemic surveillance are expected to have the highest impact, while mobile health platforms and digital therapeutics achieve the highest feasibility scores. This visualization highlights the trade-offs between cost, impact, and implementation practicality, which form the basis for the Investment Priority Score calculations.

**Figure 3:**
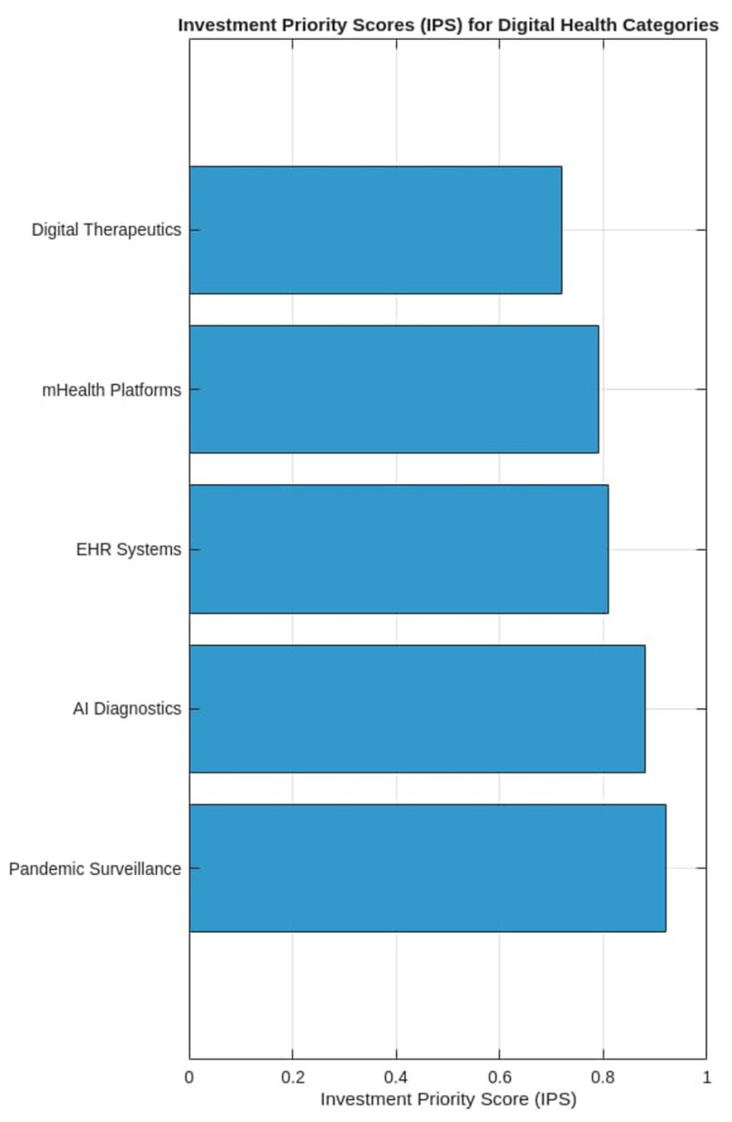
Investment Priority Scores (IPSs) for digital health categories (researchers’ findings).

Figure 3 displays the Investment Priority Scores (IPSs) derived from fuzzy logic (Table 3), ranking digital health investments from highest to lowest priority.

Pandemic surveillance ranks highest, followed by AI diagnostics and EHR systems. The chart illustrates the relative contribution of each investment to system resilience and emergency preparedness, providing policymakers with a clear and accessible tool for priority setting.

**Figure 4:**
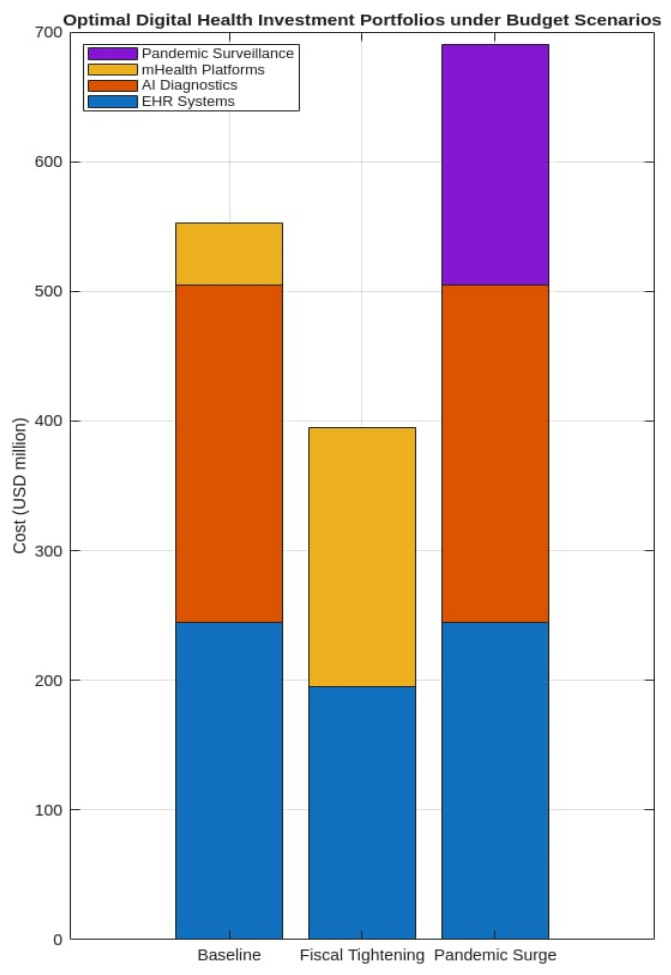
Optimal investment portfolios under three budget scenarios (researchers’ findings).

Figure 4 illustrates the total Investment Priority Scores (IPSs) achieved under baseline, fiscal tightening, and pandemic surge scenarios (Table 4).

The combination of line and stacked bar charts highlights how varying budget constraints influence portfolio composition and overall system efficiency. Notably, pandemic surge scenarios prioritize surveillance and AI diagnostics, yielding the highest total IPS of 2.61. In contrast, fiscal tightening reduces overall priority scores, underscoring the trade-off between budgetary limits and resilience optimization.

**Figure 5:**
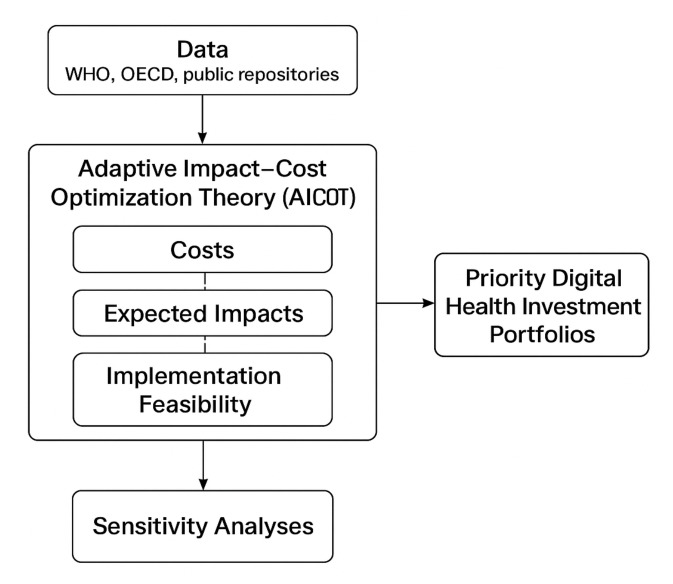
Adaptive impact–cost optimization framework for digital health investment prioritization.

The flowchart in Figure 5 presents the structured workflow of the Adaptive Impact–Cost Optimization Theory (AICOT) model, designed to prioritize digital health investments.

Data Sources: The process begins with the collection of empirical data from authoritative sources, including the World Health Organization (WHO) and the Organization for Economic Co-operation and Development (OECD), as well as other publicly available repositories. These datasets provide comprehensive information on health outcomes, costs, and infrastructure, forming the empirical foundation necessary to inform the model.

Adaptive Impact–Cost Optimization Theory (AICOT): Within the AICOT framework, three core components are systematically evaluated: Costs, Expected Impacts, and Implementation Feasibility. The Costs component quantifies both direct and indirect expenditures associated with each digital health intervention. The Expected Impacts component estimates anticipated benefits, including improvements in health outcomes and system efficiency. Finally, Implementation Feasibility assesses operational and contextual factors such as institutional capacity and technological readiness that influence the practicality of deploying each intervention.

Priority Investment Portfolios (Budgets): By integrating these components, the AICOT model generates Priority Investment Portfolios. These portfolios represent optimized bundles of digital health initiatives that maximize impact while balancing costs and feasibility constraints, enabling decision-makers to allocate resources effectively within budgetary and contextual limitations.

Sensitivity Analyses: To validate the robustness of the recommendations, sensitivity analyses were conducted as a final step. These analyses assess the stability of investment priorities by examining variations in input assumptions and parameter uncertainties, ensuring that the model’s outputs remain reliable across different scenarios.

## 6. Results

The empirical findings reveal substantial differences in digital health readiness across OECD countries. Countries with stronger electronic health record coverage, higher adoption of artificial intelligence tools, and better surveillance capacity generally achieved stronger emergency response performance. These findings indicate that investment success depends not only on spending levels but also on strategic allocation and institutional readiness. Analysis of the 15 OECD countries (Table 2, Figure 1) reveals substantial heterogeneity in digital health infrastructure, expenditure patterns, and pandemic preparedness indicators. Countries such as Sweden, the Netherlands, and the United Kingdom consistently demonstrate strong performance across key metrics, including EHR coverage (over 98%), adoption of AI-enabled diagnostics (over 40%), mHealth penetration (over 50%), and readiness scores for surveillance systems (over 0.88). These high-performing nations also exhibit elevated Pandemic Digital Response Scores (>80), underscoring their relative resilience during public health emergencies. In contrast, Japan and Italy report lower adoption of AI diagnostics and mHealth applications, coupled with moderate surveillance readiness (0.70–0.72), indicating potential vulnerabilities in rapidly scaling digital health solutions during crises. The observed variation across countries reflects both historical investment trajectories and differing levels of policy prioritization for digital health within national health systems. Indeed, the COVID-19 pandemic has prompted substantial changes in national health policies, leading to the emergence of novel practices. In particular, the pandemic underscored the critical role of telemedicine, with tele-nursing emerging as a key component of remote care delivery. Post-pandemic developments in remote healthcare practices have further highlighted the importance of integrating digital technologies into routine care [66].

These patterns highlight a core principle in health economics: investment efficiency is determined not only by the magnitude of spending but also by strategically targeted allocation aligned with system priorities. Countries with higher average intervention costs (USD 260–310 million) often leverage economies of scale within integrated health systems, whereas lower-cost systems may encounter trade-offs between coverage and operational efficiency. This finding is consistent with prior research emphasizing the importance of strategic, rather than purely quantitative, digital health budgeting across OECD settings. Budgets derived from limited resources should be assessed in terms of both effectiveness and efficiency. However, since the full potential of such analyses is often not fully understood, decision making frequently relies on individual preferences rather than evidence-based evaluation [67].

The fuzzy input dataset (Table 3, Figure 2) evaluates five digital health investment categories across three critical dimensions: normalized cost, expected impact, and implementation feasibility. AI diagnostics and pandemic surveillance exhibit the highest expected impact scores (0.90 and 0.95), reflecting their substantial potential to strengthen both routine service delivery and emergency response capacity. However, AI diagnostics are associated with relatively high implementation costs (0.78) and moderate feasibility (0.62), underscoring the trade-offs policymakers encounter when prioritizing high-impact technologies. By contrast, mobile health platforms and digital therapeutics demonstrate lower costs (0.48–0.55) and higher feasibility (0.80–0.88), indicating that although these interventions are comparatively easier to deploy, their contributions to overall system resilience may be more limited.

From a health economics perspective, these trade-offs are particularly salient. The use of fuzzy logic enables decision-makers to incorporate multidimensional uncertainties into cost-effectiveness assessments, moving beyond conventional cost-per-unit analyses. By accounting for variability in implementation feasibility, the model captures real-world constraints such as workforce capacity, interoperability barriers, and institutional readiness, thereby providing a more realistic foundation for strategic investment planning. Digital transformation projects in healthcare are both costly and time-intensive to mature. As healthcare systems plan significant investments in digital transformation, total cost analysis plays a critical role in minimizing conflicts and reducing waste across healthcare and hospital services. Previous studies have demonstrated that achieving desired outcomes requires a systematic project management approach aligned with sector-specific targets, typically yielding results over the medium to long term. Total cost analysis provides valuable insights into the economic relationships among different stakeholders. Additionally, social cost–benefit analysis (SCBA) is crucial for the planning, monitoring, and evaluation of digital hospital investments. SCBA offers a comprehensive framework for assessing digital transformation initiatives, optimizing healthcare investments, and addressing complex challenges in healthcare service delivery [29,68,69].

Fuzzy inference generates Investment Priority Scores (IPSs) (Table 4, Figure 3), translating multidimensional input data into actionable rankings. Pandemic surveillance attains the highest IPS (0.92), followed closely by AI diagnostics (0.88). EHR systems and mobile health platforms receive intermediate priority scores (0.79–0.81), reflecting their dual function in sustaining routine service delivery while enhancing pandemic preparedness. Digital therapeutics obtain the lowest score (0.72), indicating a comparatively modest contribution to overall resilience optimization. These findings are consistent with those in the contemporary literature, which underscores the strategic value of integrating pandemic-specific digital infrastructures into routine health system investments [23].

The genetic-algorithm-driven optimization identifies cost-effective investment portfolios across three budget scenarios (Table 5, Figure 4):

1. Baseline Scenario (USD 600 million): A blended portfolio comprising EHR systems, AI diagnostics, and mobile health platforms achieves a total IPS of 2.48. This configuration balances routine service delivery with pandemic preparedness, demonstrating that integrating both high-impact and highly feasible interventions maximizes system-wide efficiency.
2. Fiscal Tightening Scenario (USD 400 million): Under constrained budget conditions, the model prioritizes lower-cost, high-feasibility investments (mHealth + EHR), producing a total IPS of 1.60. Although overall efficiency decreases, the portfolio maintains essential digital health functions, illustrating the adaptive capacity of AICOT in environments characterized by fiscal uncertainty.
3. Pandemic Surge Scenario (USD 700 million): In this context, resilience-critical investments—particularly pandemic surveillance and AI diagnostics dominate the optimal portfolio, generating the highest total IPS of 2.61. This outcome highlights the importance of scenario-aware budgeting, which involves making targeted investments during periods of heightened demand to significantly enhance system responsiveness.

Collectively, these findings indicate that dynamic, scenario-based investment strategies consistently outperform static allocation approaches, particularly in settings defined by uncertainty and fluctuating health system pressures. The applicability of these strategies varies depending on factors such as the healthcare system, capital structure, technological sophistication, and geographical context. Reimbursement policies, as well as the relative contributions of public and private sector investments, are key determinants of healthcare investment decisions. Successful investments are influenced by the development level of healthcare infrastructure, the availability of advanced technological capabilities, the strategic allocation of resources, and the feasibility of integrating artificial intelligence (AI) technologies. However, the high cost of AI solutions and the complexity of regulatory approval processes represent significant barriers to their widespread adoption [33].

## 7. Discussion

The findings suggest that adaptive and scenario-based budgeting approaches are more effective than static allocation methods in digital health planning. This is particularly relevant for healthcare systems operating under uncertainty, inflationary pressure, or sudden emergency demand.

The study also indicates that mixed investment portfolios provide stronger long-term value. While lower-cost technologies such as mobile health platforms support efficiency and access, higher-impact tools such as surveillance systems and AI diagnostics become essential during crisis periods. Therefore, resilient healthcare budgeting should combine routine efficiency with emergency preparedness.

Studies evaluating the cost, impact, and value of digital health interventions are critical for supporting sustainability initiatives. They play a key role in guiding decision-makers in the approval, large-scale adoption, and funding of such interventions [70]. Cost–impact curves generated by the model indicate that marginal gains in system resilience are nonlinear. High-cost interventions, such as AI diagnostics, yield substantial impact but entail steep opportunity costs. Artificial intelligence (AI) can be employed to simulate the effects of various strategies or policies, thereby supporting evidence-based decision making and enhancing the efficiency, effectiveness, and sustainability of healthcare systems [71]. In contrast, lower-cost interventions, such as mHealth platforms, provide incremental improvements at relatively minimal expenditure. Blended portfolios that integrate both high-impact and highly feasible interventions optimize the cost–impact frontier, ensuring that budgetary constraints do not disproportionately compromise system preparedness. The sensitivity analysis further demonstrates the robustness of the model under varying economic conditions. Specifically, when investment costs are increased by 10%, pandemic surveillance consistently remains the highest-priority investment due to its dominant impact on system resilience and emergency preparedness. While high-cost technologies such as AI diagnostics exhibit moderate sensitivity to cost fluctuations, lower-cost and highly feasible interventions particularly mobile health platforms gain relative importance under inflationary scenarios. Despite these shifts, the overall ranking structure remains stable, indicating that the model’s prioritization logic is not overly sensitive to parameter perturbations. This stability reinforces the reliability of AICOT as a decision-support tool under real world uncertainty, including inflationary pressures and supply chain disruptions.

Sensitivity analysis reveals specific ranking adjustments under simulated macroeconomic shocks. Under a 10% inflation scenario, AI diagnostics declined from second to fourth place in the constrained-budget portfolio due to increased implementation cost. Under a 15% supply chain disruption scenario, surveillance systems declined by approximately 8% in priority score because of procurement delays. In contrast, mobile health platforms improved from fourth to second position under both scenarios due to lower deployment cost and faster scalability.

Sensitivity analyses further reveal that factors such as inflationary pressures, fiscal tightening, or sudden surges in service demand can significantly alter the rankings of portfolios. These findings underscore the importance of adaptive, evidence-informed budgeting, in which investment decisions are continuously updated to reflect evolving economic conditions. Given the potentially high opportunity costs associated with investing in digital health interventions (DHIs), countries must carefully determine which interventions to scale. In developing countries, where economic evaluations of DHIs remain limited, making informed investment decisions requires a clear understanding of the values and priorities within the local health system. The combination of scarce resources and uncertainty underscores the need for an analytical approach to identifying and prioritizing high-value investments in the digital health sector [72]. Importantly, this observed heterogeneity is not merely descriptive but serves as an implicit validation mechanism for the AICOT framework. The model successfully differentiates between high-performing digital health systems (e.g., Sweden, the Netherlands) and moderately performing systems (e.g., Italy, Spain), reflecting variations in infrastructure maturity, digital adoption, and policy readiness. In high-adoption contexts, stronger feasibility and impact indicators translate into higher IPS values and more resilience-oriented optimized portfolios. Conversely, in medium-adoption countries, relatively lower feasibility and adoption levels result in more constrained optimization outcomes. This sensitivity to structural differences demonstrates that the model is context-aware and capable of generating differentiated policy recommendations, thereby confirming its external validity and robustness across heterogeneous health system environments. A limitation of the present framework is that investment categories are treated as independent alternatives. In practice, complementarities may exist between electronic health records and AI diagnostics through shared data infrastructure, while coordination gains may also emerge between mobile health platforms and surveillance systems. Conversely, implementation bottlenecks or competition for technical capacity may create negative interactions. Future research should therefore incorporate interaction coefficients, network effects, or system-dynamics structures.

## 8. Conclusions, Policy Implications, and Suggestions for the Future

In conclusion, this study introduces AICOT as a practical framework for improving digital health investment decisions under limited budgets. By jointly evaluating cost, expected impact, and implementation feasibility, the model provides transparent and scenario-sensitive policy guidance.

The results indicate that blended portfolios combining routine digital services with emergency-response technologies generate the strongest resilience-adjusted efficiency. Healthcare policymakers may therefore benefit from moving beyond static budgeting models toward adaptive investment planning approaches.

The Adaptive Impact–Cost Optimization Theory (AICOT) model demonstrates that adaptive, scenario-aware digital health investment strategies can substantially enhance system resilience, efficiency, and pandemic preparedness. The AICOT framework integrates country-level digital health indicators, fuzzy-logic-based uncertainty modeling, and genetic-algorithm-driven optimization to generate evidence-based, scenario-sensitive investment portfolios. By integrating country-level indicators, fuzzy logic, and optimization algorithms, the framework equips policymakers with a transparent and actionable tool for strategic resource allocation. The analysis confirms that blended portfolios, which combine routine and pandemic-focused digital tools, achieve the highest resilience-adjusted efficiency, providing a pathway toward more robust, sustainable, and equitable health systems across OECD countries. The findings highlight several critical insights. First, blended portfolios that combine routine digital health interventions—such as electronic health records (EHRs), mobile health platforms, and digital therapeutics—with pandemic-oriented infrastructures, including AI-enabled diagnostics, surveillance systems, and tele-epidemiology tools, consistently deliver the highest resilience-adjusted efficiency. Second, scenario-based budgeting enables policymakers to dynamically adapt investment decisions in response to fiscal constraints or emergency surges, optimizing cost–impact trade-offs without compromising essential services. Third, the framework introduces transparent priority setting through Investment Priority Scores, supporting the accountable allocation of limited resources in alignment with Universal Health Coverage (UHC) and the Sustainable Development Goals (SDGs). Sustainable Development Goal 3 (SDG 3), “Good Health and Well-being,” establishes key targets aimed at ensuring healthy lives and promoting well-being for all. While SDG 3 serves as the primary health-focused goal, numerous other SDGs contain targets that directly impact health outcomes, including those related to nutrition, water and sanitation, gender equality, climate action, and poverty reduction. Collectively, these goals shape the broader social and environmental determinants of health. Beyond its economic and operational contributions, the AICOT framework also aligns with broader sustainability objectives. By prioritizing cost-effective and high-impact digital health investments, the model contributes to reducing inefficiencies, minimizing unnecessary expenditures, and optimizing the use of limited public resources. These outcomes directly support Sustainable Development Goal 3 (Good Health and Well-being), particularly in terms of improving access, quality, and system resilience. Furthermore, digital health solutions such as telemedicine and remote monitoring reduce the need for physical infrastructure and patient travel, thereby generating indirect environmental benefits. In this respect, AICOT not only enhances financial sustainability but also contributes to the long-term environmental and social sustainability of public health systems. Importantly, the framework was intentionally designed to remain understandable for healthcare administrators and public-sector decision makers without requiring advanced technical expertise. These additional robustness clarifications strengthen the transparency and scalability of the proposed framework for future policy applications.

### 8.1. Limitations

This study has several limitations. While AICOT provides a robust framework, several limitations warrant consideration. Firstly, the Geographic Scope of the data is limited to OECD countries and may not be generalizable to low- and middle-income settings. Secondly, Fuzzy input parameters rely on expert judgment, which introduces potential subjectivity. Thirdly, the model is a simplified investment model for allocating the budget. The model currently evaluates discrete investment categories and does not account for complex interdependencies among digital health technologies. While the model provides a structured and transparent framework for prioritization, it currently evaluates digital health investment categories as independent entities. In practice, however, substantial interdependencies exist between technologies. For instance, interoperable Electronic Health Records (EHR) systems can significantly enhance the effectiveness of AI-based diagnostics, while mobile health platforms can amplify the reach and responsiveness of surveillance systems. The absence of these interaction effects may lead to a conservative estimation of combined benefits, indicating an important area for further model refinement.

### 8.2. Policy Implications

The results have several key implications for health economists and policymakers. Firstly, strategic portfolio planning can be for budget allocation. Investments should prioritize high-impact, resilience-enhancing interventions while accounting for feasibility and cost considerations. Secondly, this scenario-aware allocation enables AICOT to facilitate dynamic budgeting, allowing health systems to anticipate and respond effectively to fiscal constraints or pandemic shocks. Thirdly, it can establish evidence-based decision making. Transparent Investment Priority Scores (IPSs) and cost–impact visualizations enhance accountability and facilitate alignment with Universal Health Coverage (UHC) and the Sustainable Development Goals (SDGs). From a policy perspective, AICOT offers a practical and analytically robust instrument for guiding strategic digital health investments, facilitating evidence-informed decision making, enhancing system preparedness, and ensuring the efficient allocation of fiscal resources to areas with the best health and economic returns. Future applications of the framework may extend to low- and middle-income countries, incorporate more granular financial data, and assess long-term effects on health system performance. Given the complexity of digital transformation, effective planning requires models that can address uncertainty while integrating both economic and operational dimensions. AICOT directly responds to this need. Future studies are encouraged to examine its applicability in LMIC contexts further, explore long-term impact trajectories, and develop interactive decision-support systems.

### 8.3. Suggestions for the Future

Building upon the AICOT, several avenues for future research and practical application emerge. While AICOT has been applied to 15 OECD countries, future studies could extend the framework to LMICs, where digital health adoption, infrastructure maturity, and fiscal constraints differ substantially. Adapting the model to diverse health system contexts would generate context-specific investment portfolios, thereby enhancing global health equity and preparedness. Future iterations could account for interaction and inter dependencies among digital health technologies—for example, how EHR interoperability enhances the performance of AI-driven diagnostics or how mobile health (mHealth) platforms strengthen pandemic surveillance. Modeling these interactions would support more nuanced portfolio optimization and more realistic scenario simulations. Incorporating macroeconomic simulations (such as inflationary pressures, fiscal tightening, or unexpected pandemic surges) could strengthen AICOT’s scenario responsiveness. Future studies may also investigate how policy levers, including subsidies, incentive programs, and regulatory frameworks, influence the feasibility and impact of investment, thereby providing actionable guidance for governments. Leveraging advances in artificial intelligence and real-time health system data streams, AICOT could evolve into a continuous decision-support platform. Such a system would allow policymakers to adjust investment priorities dynamically in response to emerging health threats or technological innovations, supporting sustainable and resilient digital health ecosystems. Future research should explicitly incorporate interdependencies among digital health technologies to better reflect real-world system dynamics. One promising direction involves the use of multi-objective genetic algorithms (MOGA), which can simultaneously optimize cost, impact, and interaction effects across technologies. Additionally, network-based modeling approaches such as graph theory can be employed to simulate synergistic relationships, where the value of one investment depends on the presence of another. Integrating these approaches would allow the AICOT framework to move beyond independent evaluation toward a systems-level optimization perspective, potentially yielding higher resilience-adjusted efficiency and more realistic policy recommendations.

## Figures and Tables

**Table 1:** Research findings from health economics, digital systems research, and digital health investment.

Author/Year	Study Aim	Methodology	Data Source/Sample	Key Findings	Relevance/Contribution to Literature or This Study (AICOT)
El Arab & Al Moosa (2025) [33]	To examine the cost-effectiveness, benefits, and budget impact of clinical artificial intelligence (AI) interventions in various healthcare settings.	Conceptual analysis	Nineteen studies covering oncology, cardiology, ophthalmology, and infectious diseases	It demonstrates that artificial intelligence increases diagnostic accuracy, improves quality-adjusted life years, and reduces costs.	Static models fail to capture the adaptive learning of artificial intelligence systems over time; dynamic modelling, on the other hand, captures comprehensive cost elements and long-term sustainable value.
Federici et al. (2025) [54]	The assessment and comparison of costs associated with vaccination using digital health systems such as electronic immunization records (eIRs) and electronic logistics management information systems (eLMIS).	Qualitative data analysis	275 facilities	The implementation of electronic systems raised costs in both Honduras and Rwanda, lowered costs in Tanzania, and showed no significant impact on costs in Guinea.	The findings make clear that digital health solutions generate different economic impacts depending on the strength of infrastructure, the quality of implementation, and their integration within existing health systems.
Kraus et al. (2023) [55]	Effectively estimating the cost of treatments	Machine Learning and Optimization Techniques	89,191 health records	The model could save the USA USD 1.1 billion annually.	It supports the idea that healthcare management can prevent illness at a lower cost in the decision-making process.
De Vos et al. (2022) [56]	Determining the most appropriate time and cost for discharging a patient from the pacmed-critical (PC) unit	Machine Learning, Cost-Effectiveness Analysis, and Markov Models	1000 Adult patients	PC is 92% more likely to be cost-effective than SC in the Netherlands.	The study demonstrates that machine learning can be used in cost-effectiveness analyses.
Fagherazzi et al. (2022) [57]	To evaluate mHealth interventions for chronic disease management	Meta-analysis	72 randomized controlled trials	mHealth improves both clinical outcomes and cost-effectiveness	Supports AICOT’s investment category of mobile health platforms
OECD (2021) [58]	To compare digital health maturity across countries	Comparative data analysis	Digital health indicators from 38 OECD countries	Higher EHR adoption, e-prescription rates, and system interoperability are linked to improved efficiency.	Serves as a benchmark for the 15-country OECD dataset used in AICOT
WHO (2021) [23]	To evaluate the alignment of digital health investments with global goals	Policy framework analysis	Digital health strategies from 130+ WHO member states	Digital infrastructure is essential for achieving UHC and SDG health targets	Aligns with AICOT’s goal of supporting UHC- and SDG-oriented digital budgeting decisions
Agarwal et al. (2021) [19]	To quantify the economic value of hospital digitalization	Economic modeling	Financial data from digitally transforming hospitals	Digital investments reduce operational expenditures by 10–15%	Supports AICOT’s cost–impact analysis framework by demonstrating measurable efficiency gains
Keesara et al. (2020) [12]	To examine how COVID-19 accelerated digital transformation in health systems	Conceptual analysis	U.S. and OECD health system responses during COVID-19	Telehealth, digital therapeutics, and remote monitoring became essential for maintaining service continuity	Supports AICOT’s premise that mixed portfolios of routine and emergency-specific digital tools strengthen system resilience
Budd et al. (2020) [10]	To assess digital epidemiology tools for pandemic control	Systematic evidence review	95 studies on real-time tracking and tele-epidemiology	Digital surveillance improves early detection and emergency response	Strengthens AICOT’s inclusion of real-time surveillance and tele-epidemiology tools
Whitelaw et al. (2020) [9]	To assess the role of digital health technologies in pandemic management	Systematic review	Digital interventions implemented across 17 countries	AI-based screening, digital surveillance, and mobile tracking were effective in early outbreak control	Provides theoretical justification for including pandemic-specific digital infrastructure in AICOT
Topol (2019) [34]	To analyze the clinical and economic impacts of AI in healthcare	Narrative review	Global evidence on AI-enabled diagnostics	AI improves diagnostic accuracy and may yield significant cost savings	Provides economic justification for AI-based diagnostics as an investment category in AICOT
Oderanti et al. (2016) [59]	To compare budgeting methods under uncertainty	Bayesian decision theory + fuzzy modeling	Fiscal and health expenditure data from European countries	Simulation-based budgeting outperforms traditional deterministic methods during high uncertainty	Provides direct methodological support for AICOT’s fuzzy inference system

**Table 2:** Sample dataset for AICOT model (digital health indicators, 15 OECD countries, 2018–2024) (researchers’ findings).

Country	Digital Health Expenditure (% of Total Health Expenditure)	EHR Coverage (%)	AI Diagnostics Adoption (%)	mHealth Use (%)	Surveillance System Readiness Index (0–1)	Pandemic Digital Response Score (0–100)	Avg. Cost of Intervention (USD Million)
Canada	7.2	94	32	48	0.82	78	245
Germany	6.5	90	28	41	0.79	74	260
UK	8.1	98	40	52	0.88	82	230
France	6.8	92	30	45	0.81	75	255
Italy	5.9	85	22	38	0.72	68	215
Spain	6.3	89	25	42	0.75	70	220
Sweden	8.5	99	45	55	0.91	88	240
Norway	8.0	97	38	51	0.89	85	235
Japan	5.7	84	18	36	0.70	65	210
Korea	6.9	93	35	49	0.87	80	250
Australia	7.5	96	33	47	0.86	82	238
Netherlands	8.3	98	42	53	0.90	87	242
Belgium	6.4	90	27	44	0.77	73	225
Switzerland	7.8	97	34	50	0.88	84	265
USA	9.1	96	48	59	0.92	89	310

**Table 3:** Fuzzy Input Dataset for investment categories (researchers’ findings).

Investment Category	Cost (0–1)	Expected Impact (0–1)	Feasibility (0–1)
EHR Systems	0.65	0.82	0.78
AI Diagnostics	0.78	0.90	0.62
Digital Therapeutics	0.55	0.75	0.80
Mobile Health Platforms	0.48	0.72	0.88
Pandemic Surveillance & Tele-Epidemiology	0.70	0.95	0.68

Note: Values presented in Table 3 are derived from a combination of normalized OECD indicators and fuzzy-transformed expert evaluations under scenario-based weighting.

**Table 4:** Fuzzy output: Investment Priority Scores (IPSs) (researchers’ findings).

Investment Category	Investment Priority Score (IPS, 0–1)
Pandemic Surveillance & Tele-Epidemiology	0.92
AI Diagnostics	0.88
EHR Systems	0.81
Mobile Health Platforms	0.79
Digital Therapeutics	0.72

**Table 5:** Optimal digital health investment portfolio under three budget scenarios (researchers’ findings).

Scenario	Budget (USD Million)	Selected Investments	Total Cost	Total IPS
Baseline	600	EHR + AI Diagnostics + mHealth	600	2.48
Fiscal Tightening	400	mHealth + EHR	395	1.60
Pandemic Surge	700	Pandemic Surveillance + AI Diagnostics + EHR	690	2.61 (highest)

## Data Availability

The data presented in this study are available at https://data.worldbank.org/indicator, https://www.who.int/data/gho, https://www.oecd.org/health/health-data.htm, https://osf.io, https://www.kaggle.com/datasets, https://www.icpsr.umich.edu (accessed on 10 March 2026).

## Abbreviations

The following abbreviations are used in this manuscript:

AICOT	Adaptive Impact Cost Optimization Theory
CEA	Cost-effectiveness analysis
CUA	Cost-utility analysis
EHRs	Electronic Health Records
FIS	Fuzzy Inference System
GA	Genetic Algorithm
LMICs	Low- and Middle-Income Countries
MCDA	Multi-Criteria Decision Analysis
NPS	Normalized Priority Score
PSO	Particle Swarm Optimization
SDGs	Sustainable Development Goals
UHC	Universal Health Coverage
